# Dietary Supplementation with Fermented Milk Improves Growth Performance and Intestinal Functions in Intrauterine Growth-Restricted Piglets

**DOI:** 10.3390/ani15101367

**Published:** 2025-05-09

**Authors:** Qing Yang, Lu Cui, Yang Yang, Ying Yang, Zhaolai Dai, Zhenlong Wu

**Affiliations:** 1 State Key Laboratory of Animal Nutrition and Feeding, College of Animal Science and Technology, China Agricultural University, Beijing 100193, China; qing.yang@cau.edu.cn (Q.Y.); choi1994@163.com (L.C.); yang.yang@cau.edu.cn (Y.Y.); cauvet@163.com (Y.Y.); daizhaolai@cau.edu.cn (Z.D.); 2 Tangshan Animal Husbandry Technology and Promotion Station, Tangshan 063000, China

**Keywords:** fermented milk, growth performance, intestinal health, intrauterine growth restriction, piglets

## Abstract

Intrauterine growth restriction (IUGR) commonly occurs in pigs and poses a significant challenge to the swine industry. This study investigated the effect of fermented milk on growth performance and intestinal health in IUGR-affected piglets. A total of 24 28-day-old weaned piglets with IUGR were randomly assigned to a corn-soybean basal diet (control) or a basal diet mixed with fermented milk (3:1 w/v, treatment). The results showed that fermented milk increased the average daily gain and decreased the feed-to-gain ratio (*p* < 0.05). Fermented milk increased the villus height in the duodenum and decreased the jejunal crypt depth (*p* < 0.05). Pigs in the treatment showed higher activities of lipase, α-amylase, and sucrase in the duodenum, along with an elevation in jejunal sucrase activity (*p* < 0.05). The ileal glutathione concentration was increased by the treatment (*p* < 0.05). Moreover, fermented milk upregulated the protein expression of occludin and claudin-3 while decreasing the gene expression of interleukin 1 beta, interleukin 6, and tumor necrosis factor αlpha in the jejunum (*p* < 0.05). Collectively, these results indicate that dietary supplementation with fermented milk significantly improved growth performance through the enhancement of intestinal functions in IUGR piglets, highlighting the potential of fermented milk as a nutritional strategy to improve postnatal growth in IUGR piglets.

## Simple Summary

Intrauterine growth restriction (IUGR) adversely affects survival, growth, development, and health in mammals, and it commonly occurs in pigs and causes serious economic losses to the swine industry. Fermented milk, a dairy product with high nutritive value and probiotic attributes, has shown gastrointestinal and general health benefits. However, the effect of fermented milk on IUGR in pigs remains unclear. This study revealed that dietary supplementation with fermented milk increased growth rates and feed efficiency in weaned piglets with IUGR. The fermented milk also promoted intestinal development, increased digestive enzyme activities, enhanced barrier functions, and alleviated oxidative stress and inflammation in the small intestine. Overall, fermented milk improved the growth performance and intestinal health of IUGR piglets. Our research provides a potential nutritional approach for managing IUGR in swine production.

## 1. Introduction

Intrauterine growth restriction or intrauterine growth retardation (IUGR) is a phenomenon of the impeded growth and development of embryo or fetus in mammals, which remarkably reduces neonatal survival and jeopardizes the postnatal growth and health of offsprings in the long term [1]. IUGR commonly occurs in domestic animals and can be defined as fetal or birth weight below two standard deviations from the mean for gestational age [2]. Pigs, which are genetically selected for larger litter sizes in modern intensive production systems, have the highest IUGR prevalence (approximately 15–20% of neonates) among livestock species [3]. IUGR remarkably increases neonatal piglet mortality and has prolonged adverse impacts on the growth performance, organ functions, and meat quality of pigs, resulting in huge economic losses for the swine industry [1].

The intestinal tract is a key organ affected by IUGR [4]. Intestinal dysfunctions have been implicated in IUGR pigs, such as impaired development [5,6], damaged digestion [7,8], oxidative stress [8,9,10], epithelial barrier disruption [5,6,7], and mucosal inflammation [7,9,11]. Furthermore, intestinal maldevelopment and dysfunctions in IUGR pigs are manifested at different production phases [4,7,8]. Nutritional interventions have shown positive effects on the amelioration of growth retardation and intestinal impairment in IUGR pigs [5,8,10,12].

Fermented foods, produced through controlled microbial growth and enzymatic conversions of food components, confer gastrointestinal and systemic benefits via increased nutritional value and the production of bioactive compounds [13]. The probiotic fermentation of milk improves the nutritional attributes, texture, and flavor of milk, rendering fermented milk products an effective vehicle for probiotic delivery [14,15]. While studies documented the efficacy of fermented milk in improving growth performance and intestinal health in normal-weight pigs [16,17], the effects on IUGR-affected piglets remain unexplored. Therefore, we conducted the current study to test the hypothesis that dietary fermented milk supplementation could improve growth performance and intestinal development and functions in IUGR piglets, potentially providing a novel nutritional intervention for IUGR mitigation in swine production.

## 2. Materials and Methods

### 2.1. Chemicals and Materials

Dairy whole milk powder and food-grade glucose were purchased from Feihe Dairy (Qiqihar, China) and Xiwang Group (Shandong, China), respectively. The inoculant mixture (*Lactiplantibacillus plantarum*, *Pediococcus pentosaceus*, *Bacillus subtilis*, and *Saccharomyces cerevisiae*) was provided by Schaumann Agri-Trade Co., Ltd. (Shanghai, China). DeMan, Rogosa, and Sharpe (MRS) medium and yeast extract peptone dextrose (YPD) medium were purchased from Sigma-Aldrich (St. Louis, MO, USA). TRIzol RNA extraction reagent, TRUEscript RT MasterMix, and SYBR Green qPCR Mix were bought from Aidlab Biotechnologies Co., Ltd. (Beijing, China). Antibodies against claudin-1, claudin-3, occludin, and zonula occludens-1 (ZO-1) were from Sangon Biotech (Shanghai, China), while antibodies for ZO-2 and glyceraldehyde-3-phosphate dehydrogenase (GAPDH) were from Santa Cruz Biotechnology (Dallas, TX, USA). Beyotime Biotech Inc. (Shanghai, China) provided a Radio Immunoprecipitation Assay (RIPA) lysis buffer, bicinchoninic acid (BCA) protein assay kit, and horseradish peroxidase-conjugated goat anti-rabbit immunoglobulin G (IgG). The Nanjing Jiancheng Bioengineering Institute (Nanjing, China) supplied assay kits for glucose, lactic acid, ammonia, α-amylase, lipase, sucrose, maltase, and malondialdehyde (MDA).

### 2.2. Preparation of Fermented Milk

Whole-milk powder, food-grade glucose, inoculants (*P. pentosaceus*, *L. plantarum*, *B. subtilis* and *S. cerevisae*), and water at 30 °C were mixed and fermented in a 300 L fermentation barrel (Prosyn Biotech, Guangzhou, China) using the formula and conditions provided in Table S1. Briefly, the inoculum was activated in 250 mL of sterile water (30 °C) for 30 min with stirring. Whole-milk powder and glucose were dissolved in sterile water (30 °C) and subsequently inoculated with the activated starter culture for a 13.5 h fermentation at 30 °C. Samples of fermentates and the mixture before fermentation were freshly collected for microbial enumeration by triplicate serial dilution plating on an MRS agar and YPD agar, with incubation at 37 °C for 24 h, after which colonies in the agar plates were counted. The number of microorganisms was calculated and quantified as colony-forming units per liter of milk (CFU/L). Concentrations of glucose, lactic acid, and ammonia in the initial fermentation mixture and fermentates were measured using commercial assay kits according to respective instructions, while pH was measured using a calibrated pH meter (PHBJ-260, Lei-Ci, Shanghai, China). The non-fermented milk was prepared using the same formulation described in Table S1 but without fermentation. The fermented milk and non-fermented milk were prepared daily and stored at 4 °C. The contents of dry matter and crude protein in the fermented and unfermented milk were analyzed according to the Association of Official Analytical Chemists’ (AOAC) methods [18]. For amino acid analysis, approximately 2 mL of the samples was centrifuged at 3000× *g* for 10 min, and the intermediate layer of each sample was collected and stored at −20 °C.

### 2.3. Animals and Dietary Treatments

The animal trial was conducted on a commercial pig farm (Yinfamuye, Xinzheng, China). All animal procedures were approved by the Animal Care and Use Committee of China Agricultural University and complied with the Guide for the Care and Use of Laboratory Animals [19]. Twenty-four Duroc × Landrace × Yorkshire weaned piglets of mixed gender (28-day-old, body weight [BW] = 3.74 ± 0.07 kg, half female and half castrated male piglets), with birth weights less than 1.1 kg [3], were used in the animal trial. The IUGR piglets, selected from different litters of 24 healthy sows with similar parity (second or third), were weaned at 28 days old and randomly assigned into one of two groups, with 6 replicates per group and 2 mixed-gender piglets per replicate (cage). Each pen (1.2 m × 0.70 m × 0.85 m) was equipped with a feeder and a nipple drinker. Pigs in the treatment group were fed with a corn- and soybean meal-based basal diet mixed with fermented milk (3:1, w/v), while the control group received a basal diet mixed with non-fermented milk at an identical ratio. The fermented milk and non-fermented milk diets were freshly prepared. After two weeks, pigs in the two groups were fed with the basal diet from days 15 to 19 and then euthanized for sample collection.

The feed intake of each group was recorded daily, and pigs were weighed individually on days 0, 7, 14, and 19 to calculate the average daily gain (ADG), average daily feed intake (ADFI), and feed-to-gain ratio (F:G). The fecal consistency of each pen was observed and rated daily using a 4-category scoring system (1 = firm and shaped; 2 = soft and shaped; 3 = loose; and 4 = watery), and diarrhea (score ≥3) was determined as described [20,21]. The basal diet was formulated to meet the nutrient requirements of piglets, guided by the National Research Council [22], and the composition was analyzed using AOAC methods [18] (Table S4). Diets were provided *ad libitum* three times daily (06:00, 12:00, and 18:00), and standard management was offered throughout the experiment. The ambient temperature of the pig house was maintained at 24 °C to 26 °C, and relative humidity was controlled at 55–65%.

### 2.4. Sample Collection

At the end of the animal trial, blood samples from all piglets were collected via anterior vena cava puncture at 08:00 using sodium heparin vacutainer tubes and subsequently centrifuged at 3000× *g* for 15 min at 4 °C to obtain plasma for amino acid profiling. After blood sample collection, one randomly selected pig in each replicate was anesthetized with tiletamine-zolazepam (Zoletil, Lab Animal Technology Develop Co., Beijing, China) and euthanized via exsanguination [23]. Pigs were dissected, and the intestine was removed from the abdomen immediately post-euthanasia. Segments of the duodenum, jejunum, and ileum were collected and gently flushed with ice-cold phosphate-buffered saline to remove digesta.

Sections measuring about 2 cm from the distal duodenum, mid-jejunum, and mid-ileum were fixed in 4% paraformaldehyde for histological observations, whereas another 1 cm of each segment was collected into a 1.5 mL microcentrifuge tube (NEST Biotechnology Co. Ltd., Wuxi, China) for the analyses of tight junction proteins, antioxidant-related markers, and cytokines. Intestinal mucosa was aseptically scraped from the remaining sections of the duodenum, jejunum, or ileum for the measurement of digestive enzyme activity. Moreover, approximately 1 g of liver tissue was sampled into a 1.5 mL microcentrifuge tube for antioxidant capacity evaluation. All samples, except those fixed in 4% paraformaldehyde, were immediately snap-frozen in liquid nitrogen and stored at −80 °C until analyses.

### 2.5. Analysis of Amino Acid Profiles in Milk and Plasma

Amino acid analysis was performed using a validated high-performance liquid chromatography (HPLC) protocol with modifications [24]. For the quantification of free amino acids, 50 μL aliquots of defatted milk or plasma samples were mixed with 200 μL of 1.5 mol/L HClO_4_ in a 1.5 mL microcentrifuge tube, incubated at 4 °C for 30 min, and then centrifuged at 21,000× *g* for 10 min. The supernatant was transferred to a new tube for neutralization with 100 μL of 2 mol/L K_2_CO_3_, followed by centrifugation at 21,000× *g* for 10 min. A 50 μL aliquot of the supernatant was collected and diluted with 300 μL of HPLC-grade water. Chromatographic separation was performed on a Waters HPLC system (Milford, MA, USA) equipped with a C18 guard column (20–40 μm, 50 mm × 4.6 mm) and analytical column (3 μm, 150 mm × 4.6 mm), with detection by a Model 2475 Multi λ fluorescence detector.

To analyze total amino acids in the fermented milk and unfermented milk, 0.5 mL of defatted milk samples was mixed with 6 mL of 6.5 mol/L HCl in a 15 mL Kimax glass tube, sonicated for 10 min, purged with N_2_ for 1 min, and hydrolyzed at 110 °C for 24 h. The hydrolysate was then transferred to a 50 mL tube and diluted to 50 mL with H_2_O, followed by HPLC analysis. For tryptophan measurement, 50 μL of the samples was mixed with 40 mg of hydrolyzed potato starch, 950 μL of 4.2 mol/L NaOH, and 20 μL of n-octanol in a 2 mL tube; sonicated for 10 min; purged with N_2_ for 30 s; and hydrolyzed at 105 °C for 20 h. Solutions were centrifuged at 21,000× *g* for 1 min to obtain supernatants for further HPLC analysis, following the procedures as described [24].

### 2.6. Examination of Intestinal Morphology

Intestinal morphology was assessed through hematoxylin and eosin (H&E) staining following an established protocol [25]. Briefly, segments fixed in 4% paraformaldehyde were washed, sequentially dehydrated, embedded in paraffin, and sectioned into 5 µm slices using a Leica RM2235 microtome (Nussloch, Germany). Tissue sections (5 slides per sample) were deparaffinized in xylene, rehydrated in a gradient of ethanol, and stained in hematoxylin and subsequent eosin, followed by dehydration. Morphometric analysis was performed on intact, well-oriented villus-crypt units (10 units per sample) using an Olympus BX51 microscope (Tokyo, Japan) at 100× magnification. Villus height (from the tip of the villus to the crypt-villus junction) and crypt depth (from the crypt-villus junction to the base of the crypt) were vertically measured and quantified using Image Pro Plus (Rockville, MD, USA), from which the ratio of the villus height to crypt depth was calculated.

### 2.7. Determination of Digestive Enzyme Activities

Approximately 0.5 g of an intestinal mucosa sample was homogenized in 4.5 mL of ice-cold physiological saline using a tissue homogenizer. The resulting homogenates were centrifuged at 3500× *g* for 10 min at 4 °C to obtain a supernatant, and the protein concentration was determined using the BCA assay. Enzymatic activities of α-amylase, lipase, sucrase, and maltase were evaluated with commercial assay kits following the manufacturer’s instructions.

### 2.8. Quantification of Glutathione (GSH), Glutathione Disulfide (GSSG) and MDA

A frozen intestinal segment or liver sample (approximately 100 mg) was pulverized in liquid nitrogen and homogenized with a glass homogenizer. The homogenates were mixed with 1.5 mL of freshly prepared extraction solution (12 mM iodoacetic acid: 1.5 M HClO_4_ = 1:1) and then mixed with 0.75 mL of 2 M K_2_CO_3_. The resulting mixture was centrifuged at 600× *g* for 5 min, and the supernatant was afterward subjected to HPLC analysis for the measurement of contents of GSH and GSSG, as previously described [26].

For MDA quantification, approximately 0.5 g of an intestine or liver tissue sample was homogenized in 2 mL of ice-cold physiological saline and then centrifuged at 4000× *g* for 10 min at 4 °C. The supernatant was carefully collected, and the protein concentration was measured, followed by the analysis of MDA using the commercial assay kit according to the manufacturer’s protocol.

### 2.9. Quantitative Real-Time Polymerase Chain Reaction (qPCR) Analysis

The total RNA was extracted from intestinal samples using TRIzol RNA extraction reagent and quantified with Implen NanoPhotometer P330 (Schatzbogen, Munich, Germany). cDNA synthesis was carried out in a 20 μL reaction volume containing 4 μL of TRUEscript RT MasterMix, 10 μL of RNA (200 ng/μL), and 6 μL of RNase-free water using an Arktik Thermal Cycler (Thermo Scientific, Waltham, MA, USA). Quantitative PCR analysis was performed using an SYBR Green qPCR Mix on an ABI 7500 Real-Time PCR System (Applied Biosystems, Alameda, CA, USA) with the following thermal profile: initial denaturation at 95 °C for 2 min, followed by 40 cycles of 95 °C for 15 s and 60 °C for 34 s. Gene expression levels were normalized to GAPDH and calculated using the 2^−ΔΔCt^ method [27]. Primer sequences for target genes and the housekeeping gene were provided in Table S5.

### 2.10. Western Blot Analysis

Intestinal samples (50 mg) were homogenized in an RIPA lysis buffer, and the protein concentration was determined using the BCA assay. Equal amounts of protein (40 µg) were separated by 10% SDS-PAGE and transferred to PVDF membranes (MilliporeSigma, Burlington, MA, USA). After blocking in 5% skim milk for 1 h at room temperature, membranes were incubated with a primary rabbit antibody against claudin-1, claudin-3, occludin, ZO-1, ZO-2, or GAPDH (1:2000 dilution) in the blocking buffer for 1 h at room temperature. After washing, membranes were incubated with horseradish peroxidase-conjugated goat anti-rabbit IgG (1:5000 dilution) at room temperature for 1 h. Protein bands were visualized using an Image Quant LAS 4000 mini system (GE Healthcare, Piscataway, NJ, USA), and the band intensity was quantified using ImageJ software (version 1.54, NIH, Bethesda, MD, USA). Target protein expression levels were normalized to GAPDH as the internal control.

### 2.11. Statistical Analysis

Statistical analysis and visualization were performed using Prism (version 8.0, GraphPad Software Inc., La Jolla, CA, USA) and SAS software (version 9.4, SAS Inc., Cary, NC, USA). The results were expressed as means ± standard deviation (SD), and significance was determined by an unpaired *t*-test, with the significance set at *p* < 0.05 and trend set at 0.05 ≤ *p* < 0.10.

## 3. Results

### 3.1. Composition of the Fermented Milk

The inoculants used for fermenting milk herein consisted of *P. pentosaceus* (4.0 × 10^7^ CFU/L), *L. plantarum* (2.0 × 10^7^ CFU/L), *B. subtilis* (2.7 × 10^6^ CFU/L), and *S. cerevisae* (1.1 × 10^7^ CFU/L). The fermentation process greatly augmented the growth of these microorganisms, resulting in *P. pentosaceus* being 1.4 × 10^10^ CFU/L, *L. plantarum* being 1.3 × 10^10^ CFU/L, *B. subtilis* being 6.0 × 10^7^ CFU/L, and *S. cerevisae* being 1.1 × 10^9^ CFU/L in the fermentates (Table 1).

The fermentation resulted in a significant decrease in the content of glucose (*p* < 0.01) but a remarkable increase in lactic acid (*p* < 0.01). A decline in milk pH was also observed after fermentation (*p* < 0.01). However, the concentrations of dry matter, crude protein, and ammonia in milk were not altered by fermentation. Alternations in amino acid profile were noted in the fermented milk (Tables S2 and S3). Fermentation increased free amino acids, including alanine, aspartate, histidine, isoleucine, leucine, lysine, methionine, phenylalanine, tyrosine, citrulline, and ornithine, while decreasing glutamate levels (*p* < 0.05 or *p* < 0.01). For the total amino acids, the concentrations of arginine, isoleucine, lysine and phenylalanine were decreased after fermentation (*p* < 0.05), whereas the content of other amino acids was not changed during fermentation.

### 3.2. Dietary Supplementation with Fermented Milk Improved the Growth Performance of Weaned IUGR Piglets

The 28-day-old weaned IUGR piglets were fed with a basal diet (19% crude protein, Table S4) mixed with fermented milk (treatment) or unfermented milk (control) for 2 weeks. At the beginning of this study, the BW of piglets in the control group and treatment group was 3.73 kg and 3.75 kg, respectively (Table 2). During the first week of dietary intervention, supplementation with fermented milk did not significantly affect the weight gain, feed intake, or feed conversion ratio in IUGR piglets compared to the unfermented milk control. However, after two weeks of treatment, the BW of piglets in the fermented milk group tended to be higher than those in the control group (*p* = 0.08). The dietary supplementation of IUGR pigs with fermented milk increased the ADG from days 8 to 14 and from day 0 to 14 post-weaning (*p* ≤ 0.05). Fermented milk also had a tendency to increase the feed intake of piglets during the second week (*p* = 0.06) and improved feed conversion efficiency throughout the feeding period (*p* < 0.01). Moreover, the incidence of diarrhea was not affected by the fermented milk during the study (*p* > 0.05, Table 2). In addition, the concentrations of arginine and glutamine were significantly higher in the plasma of pigs supplemented with fermented milk (*p* < 0.05), but the levels of other amino acids were comparable between the two groups (Table 3).

### 3.3. Fermented Milk Improved Intestinal Morphology in IUGR Piglets

**Figure 1:**
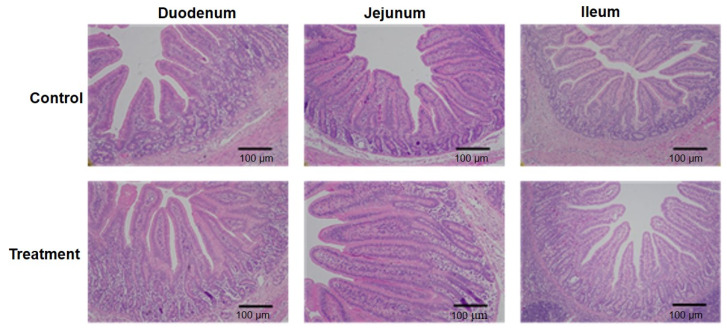
Intestinal morphology of IUGR pigs fed the basal diet supplemented with unfermented milk (control) or fermented milk (treatment). Cross-sections of the small intestine were H&E-stained and then examined under a microscope at 100× magnification.

The small intestinal segments of fermented-milk-fed IUGR piglets overall exhibited more intact villus–crypt architectures than those of IUGR piglets supplemented with unfermented milk, indicating a potential promoting effect on intestinal morphology by fermented milk (Figure 1). Numerically, the treatment with fermented milk increased villous heights and shortened crypt depths in the duodenum, resulting in an increased ratio of villous height to crypt depth (*p* < 0.05 or *p* < 0.01, Table 4). The fermented milk decreased crypt depths and increased the ratio of the villous height to crypt depth in the jejunum of piglets (*p* < 0.05). Additionally, a lower ratio of the villous height to crypt depth was observed in the ileum of IUGR pigs supplemented with fermented milk (*p* < 0.05).

### 3.4. Fermented Milk Increased Digestive Enzyme Activity in the Intestine of Weaned IUGR Pigs

Piglets supplemented with fermented milk showed higher enzymatic activities of lipase (*p* < 0.05), α-amylase (*p* < 0.05), sucrase (*p* < 0.05), and maltase (*p* = 0.08) in the duodenum, as compared with the unfermented milk group (Table 5). Jejunal sucrose activity (*p* < 0.05) and ileal maltase activity (*p* = 0.06) were enhanced in response to fermented milk, with activities of lipase and α-amylase in the jejunum and ileum not being affected by the treatment.

### 3.5. Fermented Milk Enhanced Antioxidant Capacity in Weaned IUGR Piglets

The fermented milk intervention resulted in a marked increase in the reduced-to-oxidized glutathione ratio (GSH:GSSG) in the jejunum (*p* < 0.05), despite no significant alterations in the absolute concentrations of GSH and GSSG (*p* > 0.05, Table 6). Supplementation with the fermented milk also improved GSH content and raised the GSH:GSSG ratio in the ileum (*p* < 0.01). The jejunal or ileal MDA content was comparable between the two groups. Additionally, pigs fed with fermented milk had a higher GSH:GSSG ratio but a lower MDA content in the liver than the control group (*p* < 0.01).

### 3.6. Feeding Fermented Milk Improved the Intestinal Barrier Function of IUGR Pigs

**Figure 2:**
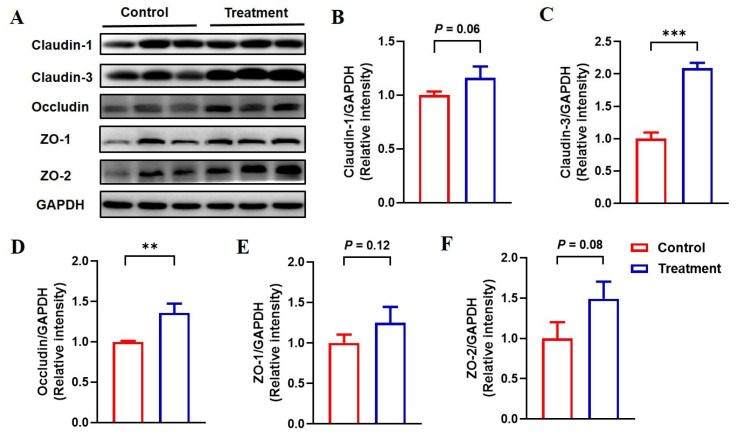
Expression of tight junction proteins in the jejunum of IUGR pigs fed the basal diet supplemented with unfermented milk (control) or fermented milk (treatment). Representative tight junction proteins in the intestine were quantified using Western blot analysis. (**A**) Western blot results of claudin-1, claudin-3, occludin, ZO-1, ZO-2, and GAPDH protein expression. (**B**–**F**) The expression of claudin-1 (**B**), claudin-3 (**C**), occludin (**D**), ZO-1 (**E**), and ZO-2 (**F**) relative to GAPDH. Data are analyzed with an unpaired *t*-test and expressed as the means ± SD, n = 6 per group. ** *p* < 0.01 and *** *p* < 0.001.

To evaluate the potential beneficial effects of fermented milk on intestinal barrier integrity in IUGR pigs, the expression levels of representative tight junction proteins were quantitatively measured using Western blot analysis (Figure 2). Dietary supplementation with fermented milk strengthened the expression of claudin-3 and occludin in the jejunum (*p* < 0.05) and tended to enhance the expression of claudin-1 (*p* = 0.06) and ZO-2 (*p* = 0.08), but it did not cause a significant change in jejunal ZO-1 expression in IUGR piglets (*p* = 0.12).

### 3.7. Dietary Fermented Milk Suppressed Intestinal Inflammation in IUGR Piglets

**Figure 3:**
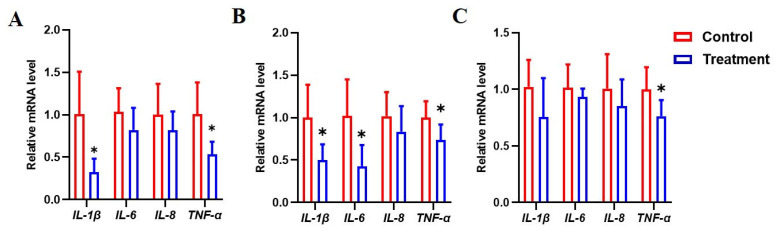
Expression of cytokine genes in the intestine of IUGR pigs fed the basal diet supplemented with unfermented milk (control) or fermented milk (treatment). The expression of proinflammatory cytokines in the duodenum (**A**), jejunum (**B**), and ileum (**C**) was determined using qPCR analysis. Data are analyzed with an unpaired *t*-test and expressed as means ± SD, n = 6. * *p* < 0.05.

To investigate whether fermented milk could modulate inflammation in the intestine, the gene expression of inflammatory cytokines was evaluated (Figure 3). In the duodenum, fermented milk reduced the gene expression of interleukin 1 beta (*IL-1β*) and tumor necrosis factor αlpha (*TNF-α*) (*p* < 0.05) (Figure 3A). The mRNA levels of *IL-1β*, interleukin 6 (*IL-6*), and *TNF-α* in the jejunum were lower in the fermented milk group in contrast with the unfermented group (*p* < 0.05) (Figure 3B). In the ileum, the supplementation of IUGR piglets with fermented milk suppressed the expression of *TNF-α* (*p* < 0.05), while the mRNA levels of *IL-1β*, *IL-6*, and *TNF-α* were not affected (*p* > 0.05) (Figure 3C).

## 4. Discussion

The restricted intrauterine growth of mammals remarkably decreases the survival probability of neonates and has permanent adverse effects on the surviving offspring [1]. IUGR commonly occurs in pigs and causes low birth weight and impaired intestines [3]. This study has revealed that dietary supplementation with fermented milk improved growth performance in contrast to non-fermented milk through the enhancement of intestinal development and functions in piglets with IUGR, providing a promising nutritional approach for the management of IUGR in swine production.

Research has demonstrated that IUGR compromises the growth performance of pigs from birth to slaughter [28,29]. A lower body weight or reduced feed efficiency was noticed in IUGR pigs at different growth phases [7,30]. In this study, feeding a diet supplemented with fermented milk for two weeks accelerated the growth rate and improved the feed utilization efficiency in IUGR piglets compared with non-fermented milk. However, the administration of fermented milk for one week was not adequate for improving the growth performance of IUGR pigs in the current scenario. Similarly, feeding lactic-acid-fermented milk for two weeks improved the growth performance of healthy weaned piglets, and the growth-promoting effect of fermented milk was not observed when it was provided for one week [17]. These consistent findings suggest that fermented milk should be administered for at least two weeks to generate positive outcomes on the growth performance of piglets. Studies have indicated that postnatal nutritional restriction exacerbated the malfunction of the intestine in IUGR piglets [31,32]. Dietary supplementation with fermented milk herein increased feed intake in the second week after weaning. The intensified nutrient intake plausibly promotes intestinal development in piglets suffering from IUGR. Moreover, the intestinal health status is closely associated with the growth performance of IUGR pigs. The impairment of intestinal morphologies and functions was implicated in IUGR pigs with reduced feed efficiency and delayed growth rates [1]. In contrast, the restored intestine was observed in IUGR pigs with catch-up growth [4]. Therefore, approaches that improve intestinal health have the potential to restore growth retardation in IUGR pigs.

The morphology of the intestine is damaged in IUGR newborn piglets, and this effect could persist in the later growth stages. Newborn IUGR piglets had shorter villi and decreased ratios of villus height to crypt depth in the jejunum compared with normal neonatal piglets [6], which was also noted in suckling piglets with IUGR [5,7,8]. Moreover, IUGR piglets have reduced intestinal length and weight, along with lower intestinal blood flow [33]. IUGR neonatal pigs had lower weights and shorter lengths with respect to the small intestine, which persisted to 70 days of age [29]. Furthermore, the damage with respect to morphology and development was prominent in the small intestine, especially in the proximal regions [5,8,29]. For example, a study reported structural impairment in the duodenum but not in the jejunum of IUGR pigs [29]. The fermented milk supplementation herein has a more pronounced beneficial impact on the improvement of the morphology of the duodenum and jejunum than that of the ileum. In healthy piglets, fermented milk significantly increased the weight and length of the small intestine rather than the large intestine and more obviously improved ileal morphology than the colon [16,17]. These results collectively indicate that fermented milk primarily stimulates the development of the small intestine in pigs irrespective of the health status.

The small intestine is critical for the digestion and absorption of nutrients, which is compromised in IUGR pigs at different production phases. IUGR decreased the activities of lactase and maltase during the suckling period [34]. The activities of digestive enzymes such as amylase, lipase, trypsin, maltase, and lactase decreased in the jejunum of IUGR-suffering weaned piglets compared with normal pigs [35]. Another study has observed diminished activities of amylase in the duodenum and weakened chymotrypsin in the jejunum of growing–finishing IUGR pigs [29]. Compared with normal-weight piglets, IUGR piglets showed lower glucose absorption capacity, which was associated with the downregulated expression of sodium-glucose linked transporter 1 (SGLT1) and Na^+^/K^+^-ATPase in the small intestinal epithelium [7]. Similarly, the expressions of amino acid transporters, glucose transporters, and fatty acid transporters in the small intestine were reduced by IUGR [4]. Our research has revealed that fermented milk increased the activity of digestive enzymes in the small intestine of IUGR piglets, with the duodenum being affected most pronouncedly, which was in line with its effect on intestinal morphological improvement. It is likely that fermented milk promoted the development of the small intestine and thus strengthened the digestive and absorptive functions of enterocytes.

Studies have demonstrated that intestinal redox homeostasis is disrupted by IUGR in pigs [9,10,36,37]. GSH, a major antioxidant rich in the small intestine and liver, is synthesized from cysteine, glutamate, and glycine with the catalysis of γ-glutamylcysteine synthetase and glutathione synthetase. GSH is oxidized to GSSG in the presence of oxidants, while GSSG is enzymatically reduced to GSH through the action of glutathione reductase with the concomitant oxidation of nicotinamide adenine dinucleotide phosphate (NADPH) to NADP^+^ [10]. Therefore, the concentrations of GSH and GSSG and the ratio of GSH to GSSG are important indicators of oxidative stress. Additionally, total antioxidant capacity (T-AOC), superoxide dismutase (SOD), catalase (CAT), and glutathione peroxidase (GSH-Px) are also important antioxidant indicators, while hydrogen peroxide (H_2_O_2_) and MDA are oxidative stress markers [38]. IUGR increased the levels of MDA and H_2_O_2_ in plasma and the liver, together with reductions in GSH-Px activity and T-AOC concentrations in the liver of newborn piglets [36]. Another study characterized oxidative stress in the liver of IUGR neonatal piglets and elucidated metabolic abnormalities associated with oxidative stress [38]. In suckling piglets, IUGR reduced the concentration of T-AOC and downregulated the activities of GSH-Px, SOD, and CAT in serum and the jejunum [7]. The oxidative damage caused by IUGR persisted in the later growth stage of pigs. The production of T-AOC and the activity of SOD were reduced, while MDA was enriched in the plasma and colon of growing–finishing pigs with IUGR [9]. In IUGR finisher pigs, the GSH content, GSH to GSSG ratio, and the activity of GSH-synthetic enzymes were reduced in various tissues, such as plasma, liver, jejunum, and muscles [10]. In the current study, supplementation with fermented milk increased the ratios of GSH to GSSG in the jejunum, ileum, and liver of IUGR pigs relative to non-fermented milk. Fermented milk increased the amount of glutamine in plasma, which can be converted to glutamate and then metabolized into GSH. Fermented milk probably enhanced the activities of GSH-synthetic enzymes in the intestine or liver to promote the synthesis of GSH. Moreover, fermented milk reduced the concentration of MDA in the liver. These data consistently suggest that fermented milk alleviated oxidative stress in IUGR pigs.

Maintaining intestinal integrity is crucial for health, as intestinal epithelial barriers prevent the translocation of pathogens and toxins. However, intestinal barrier dysfunctions have been implicated in IUGR pigs at different growth stages [9,11]. For example, the expressions of ZO-1 and occludin were attenuated in the colon of IUGR piglets aged 7 days and 21 days compared with normal-weight piglets [11]. Suckling piglets with IUGR had a lower expression of claudin-1 and ZO-1 in the jejunum than normal piglets [5,7]. Growing–finishing pigs with IUGR reduced the expression of tight junction proteins such as occludin, claudin-1, and ZO-1 in the colon when compared with counterparts with normal weight [9]. We found that fermented milk significantly increased the expression of claudin-3 and occludin in the jejunum of weaned piglets with IUGR compared with non-fermented milk, indicating improved intestinal integrity due to the fermented milk.

Pigs with IUGR have been associated with inflammation in the intestine. Proinflammatory cytokines such as IL-1β and TNF-α were upregulated in the jejunum or colon of intrauterine growth-restricted piglets, in contrast with normal pigs [7,9,11]. IUGR upregulated the expression of proinflammatory cytokines in the duodenum, jejunum, and ileum of piglets, with the duodenum being severely influenced [4]. It has been demonstrated that fermented food exerts intestinal and general health benefits at least partially through the modulation of immune responses [13]. Studies reported that lactic acid bacteria-fermented milk modulated the production of TNF-α, IL-10, and IL-6 in the ileum and colon of normally weaned piglets [16,17]. Here, we similarly observed that fermented milk, in comparison to non-fermented milk, decreased the expression of IL-1β, IL-6, or TNF-α in the duodenum, jejunum, and ileum of weaned piglets with IUGR. These findings confirm the important role of fermented milk in the regulation of intestinal inflammation in pigs.

An inadequate supply of nutrients to the fetus, particularly amino acids, is a major cause of IUGR in pigs [39,40]. Lower amounts of amino acids were detected in IUGR placenta (asparagine, leucine, lysine, phenylalanine, threonine, tyrosine, and valine) [41], fetuses (arginine, glutamine, citrulline, proline, leucine, isoleucine, and histidine) [42], and postnatal piglets (aspartate, glutamate, glutamine, and isoleucine) [43]. In the current study, higher concentrations of arginine and glutamine were found in the plasma of IUGR piglets treated with fermented milk than in the counterpart piglets fed with non-fermented milk. Arginine is a functional amino acid for pregnant sows, and dietary supplementation with arginine has effectively improved embryonic and fetal survival and development [40,44]. Arginine is an essential amino acid for neonatal piglets, and its deficiency causes the growth retardation and impaired development of various organs [45]. In IUGR piglets, the enteric synthesis of arginine is disrupted due to the impairment of intestinal functions [45]. Notably, the fermentation of milk dramatically increased the concentration of citrulline, which could be converted to arginine after intestinal absorption as a precursor for endogenous arginine synthesis in pig enterocytes [45]. Glutamine is also important for fetal nutrition and growth [39]. Maternal supplementation with glutamine increased the average birth weight, reduced within-litter birth weight variations, and alleviated intestinal impairment in IUGR piglets [43]. In addition, glutamine supplementation improved intestinal morphology and integrity in weanling piglets [46]. The increased levels of arginine and glutamine observed in the current study might contribute to the amelioration of IUGR.

Lactic acid bacteria, bacilli, and yeasts are commonly used microorganisms for fermented food production. In the present study, *L. plantarum*, *P. pentosaceus*, *B. subtilis*, and *S. cerevisiae* were used to ferment milk and were enriched in fermentates. These strains have been identified as probiotics or have shown probiotic characteristics and health effects on growth and intestinal health in animals. Studies have reported the positive impacts of several probiotics on the alleviation of IUGR in pigs. For example, *Lactobacillus amylovorus* treatment enhanced the growth performance of IUGR piglets by strengthening the development and barrier function of the intestine [5]. *B. subtilis* supplementation increased feed utilization efficiency, promoted intestinal development, and enhanced the activities of maltase and sucrase in IUGR piglets [34]. Another study revealed that the oral gavage of *B. subtilis* improved the barrier disruption, oxidative stress, and excessive apoptosis of epithelial cells in the jejunum of suckling piglets affected by IUGR [47,48]. The administration of *Clostridium butyricum* improved growth performance and lipid metabolism in IUGR suckling piglets [12]. *L. plantarum*, a versatile lactic-acid-producing probiotic, could be used to alleviate inflammatory bowel diseases through the modulation of immune response, the alleviation of oxidative stress, the enhancement of epithelial barrier function, and the regulation of intestinal microbiota [49]. *P. pentosaceus* belongs to lactic acid bacteria and has been explored as a promising probiotic candidate for applications in food and feed. *P. pentosaceus* has exerted multifarious beneficial properties, such as improving food flavor, promoting animal growth, modulating inflammation and antioxidant activity, and facilitating the utilization of nutrients and antibacterial action [50]. *S. cerevisiae* is a prominent yeast probiotic that has been applied in livestock production. The supplementation of pigs with *S. cerevisiae* had positive effects on increasing feed intake, improving growth performance, promoting intestinal development, and reducing post-weaning diarrhea [51]. This study did not observe a significant difference in diarrhea incidence between fermented milk supplementation and non-fermentation groups, which is probably due to the low diarrhea rate in this small-scale study. Given the aforementioned positive outcomes of *L. plantarum*, *P. pentosaceus*, and *S. cerevisiae*, it is likely that these microorganisms have effects on IUGR, albeit further investigation is needed.

In addition to the microorganisms utilized to ferment milk, metabolites and bioactive compounds generated during the fermentation process also exert health-promoting properties. Fermented dairy products made by lactic acid bacteria produce flavor compounds, aroma enhancers, and organic acids to improve the palatability and texture of food [52], which is in line with the observation in this study that the feed intake was increased in piglets supplemented with fermented milk. Lactic acid provides better texture, facilitates preservation, and intensifies the flavor of dairy products. Consistently, the current study exhibited that the fermentation of milk raised the concentration of lactic acid when compared with unfermented milk. Other flavor compounds might possibly increase the palatability of fermented milk and its supplemented feed and therefore promote feed intake in IUGR pigs, which needs further identification and quantification. The fermentation of milk could produce bioactive peptides (e.g., casein peptides) through the microbial enzymatic hydrolysis of milk proteins. These peptides have manifold health-promotion effects, such as immunomodulatory, antioxidant, and antimicrobial properties, and exhibit therapeutic potential in inflammatory bowel disease, cardiovascular disorders, diabetes, and cancers [14,15]. Notably, peptides produced during fermentation can be affected by strains used for fermentation, protein types in milk, and fermentation conditions. Further research on purification, identification, and assessment can foster our understanding of whether and how peptides derived from fermented milk contribute to improving the growth performance and intestinal health in IUGR-affected pigs. Moreover, ingested fermentation microorganisms might further convert food or diet ingredients in vivo into biologically functional substances such as peptides, bacteriocins, amino acids, fatty acids, and organic acids, which might have effects on the gut health of the host. In addition, exopolysaccharides, another group of bioactive products derived from fermented milk, have anti-inflammatory and oxidative-stress-protective attributes [14]. Overall, substances in the fermented milk responsible for the improvement of the intestinal health of IUGR pigs are not fully understood and have not yet been characterized by further research.

## 5. Conclusions

Compared with non-fermented milk, dietary supplementation with fermented milk improved the growth performance and promoted the intestinal development and functions of weaned piglets affected by IUGR. Fermented milk might be a promising nutritional strategy to alleviate IUGR in pig production.

## Figures and Tables

**Table 1:** Composition of the fermented milk ^1^.

Item	Before Fermentation	After Fermentation	*p*-Value
*Pediococcus pentosaceus*, CFU/L	4.0 × 10^7^	1.4 × 10^10^	—
*Lactiplantibacillus plantarum*, CFU/L	2.0 × 10^7^	1.3 × 10^10^	—
*Bacillus subtilis*, CFU/L	2.7 × 10^6^	6.0 × 10^7^	—
*Saccharomyces cerevisae*, CFU/L	1.1 × 10^7^	1.1 × 10^9^	—
Dry matter, %	18.72 ± 0.05	19.04 ± 0.21	0.13
Crude protein, g/L	38.51 ± 1.30	40.12 ± 1.28	0.25
Glucose, mmol/L	207.90 ± 12.64 ^a^	184.30 ± 5.72 ^b^	<0.01
Lactic acid, mmol/L	10.28 ± 1.09 ^a^	33.60 ± 5.54 ^b^	<0.01
Ammonia, mmol/L	0.14 ± 0.02	0.18 ± 0.05	0.46
pH	6.51 ± 0.02 ^a^	5.72 ± 0.10 ^b^	<0.01

^1^ The number of microorganisms in the inoculants was expressed as the mean of triplicate data, while other parameters were expressed as mean ± SD of triplicate. Different superscripts within rows denote statistical significance at *p* < 0.05 determined by an unpaired *t*-test.

**Table 2:** Growth performance and incidence of diarrhea of IUGR weaned piglets supplemented with fermented milk or unfermented milk ^1^.

Item	Control	Treatment	*p*-Value
BW, kg			
Day 0	3.73 ± 0.28	3.75 ± 0.42	0.91
Day 7	4.60 ± 0.35	4.78 ± 0.59	0.40
Day 14	6.25 ± 0.48	6.75 ± 0.87	0.08
Day 19	7.59 ± 0.73	8.00 ± 0.87	0.23
ADG, g/d			
Days 0 to 7	125.43 ± 38.11	146.29 ± 41.57	0.20
Days 8 to 14	235.14 ± 38.11 ^a^	276.43 ± 58.89 ^b^	0.05
Days 0 to 14	180.10 ± 27.02 ^a^	216.14 ± 41.57 ^b^	<0.05
Days 0 to 19	209.29 ± 32.22	235.57 ± 41.57	0.11
ADFI, g/d			
Days 0 to 7	186.47 ± 19.11	185.61 ± 26.94	0.93
Days 8 to 14	344.29 ± 36.74	378.20 ± 13.47	0.06
Days 0 to 14	260.03 ± 22.05	275.22 ± 16.66	0.21
Days 0 to 19	311.35 ± 29.39	326.24 ± 16.66	0.32
F:G, g/g			
Days 0 to 7	1.50 ± 0.29	1.28 ± 0.20	0.15
Days 8 to 14	1.53 ± 0.20	1.38 ± 0.12	0.16
Days 0 to 14	1.46 ± 0.10 ^a^	1.29 ± 0.05 ^b^	<0.01
Days 0 to 19	1.46 ± 0.15	1.38 ± 0.22	0.53
Incidence of diarrhea, %			
Days 0 to 7	10.32 ± 11.44	11.11 ± 7.17	0.89
Days 8 to 14	0	0.79 ± 1.94	0.34
Days 0 to 14	3.97 ± 3.92	6.35 ± 2.45	0.23
Days 0 to 19	3.80 ± 4.16	5.26 ± 1.91	0.44

^1^ IUGR pigs in the control group and treatment group (n = 12 for BW and ADG, while n = 6 for other parameters) were fed with the basal diet supplemented with unfermented milk and fermented milk, respectively. Data are analyzed with an unpaired *t*-test and indicated as means ± SD. Different superscripts within rows mark statistical significance at *p* < 0.05. BW, Body weight; ADG, average daily gain; ADFI, average daily feed intake; F:G, feed-to-gain ratio.

**Table 3:** Concentrations of free amino acids (μmol/L) in the plasma of IUGR pigs supplemented with fermented milk ^1^.

Item	Control	Treatment	*p*-Value
Alanine	435.03 ± 44.11	447.56 ± 90.63	0.81
Arginine	148.60 ± 9.55 ^a^	169.31 ± 6.12 ^b^	<0.05
Asparagine	51.73 ± 22.54	53.18 ± 13.96	0.92
Aspartate	17.68 ± 6.61	17.03 ± 4.90	0.82
Glutamine	340.60 ± 71.04 ^a^	428.88 ± 46.54 ^b^	<0.05
Glutamate	159.73 ± 17.64	178.08 ± 39.19	0.31
Glycine	661.82 ± 18.62	676.22 ± 90.63	0.71
Histidine	88.11 ± 21.31	91.50 ± 23.03	0.80
Isoleucine	74.12 ± 13.23	67.45 ± 19.60	0.51
Leucine	208.93 ± 46.54	229.40 ± 66.14	0.56
Lysine	152.98 ± 58.79	149.05 ± 56.34	0.91
Methionine	26.65 ± 4.41	23.98 ± 5.39	0.42
Phenylalanine	48.20 ± 11.02	48.88 ± 16.90	0.94
Serine	188.88 ± 41.64	160.72 ± 8.57	0.17
Threonine	87.42 ± 31.84	64.63 ± 17.64	0.17
Tryptophan	25.43 ± 10.04	23.56 ± 5.39	0.57
Tyrosine	132.39 ± 41.64	115.32 ± 26.94	0.42
Valine	103.44 ± 26.94	117.67 ± 41.64	0.49
β-Alanine	74.13 ± 14.45	83.87 ± 10.78	0.31
Citrulline	104.45 ± 26.94	102.67 ± 31.84	0.90
Ornithine	126.94 ± 41.64	115.83 ± 41.64	0.65
Taurine	92.32 ± 34.29	81.35 ± 36.74	0.59

^1^ IUGR pigs in the control group and treatment group were fed with the basal diet supplemented with unfermented milk and fermented milk, respectively. Data are expressed as means ± SD, n = 6. Different superscripts within rows denote statistical significance at *p* < 0.05, determined by an unpaired *t*-test.

**Table 4:** Intestinal morphology in IUGR weaned piglets supplemented with fermented milk ^1^.

Item	Control	Treatment	*p*-Value
Duodenum			
Villous height, μm	371.23 ± 31.84 ^a^	420.84 ± 46.54 ^b^	<0.05
Crypt depth, μm	276.41 ± 24.25 ^a^	253.78 ± 14.70 ^b^	0.05
Villous height/crypt depth	1.38 ± 0.15 ^a^	1.68 ± 0.22 ^b^	<0.01
Jejunum			
Villous height, μm	420.87 ± 29.39	422.61 ± 29.39	0.92
Crypt depth, μm	227.42 ± 20.58 ^a^	195.88 ± 17.88 ^b^	<0.01
Villous height/crypt depth	1.94 ± 0.24 ^a^	2.25 ± 0.27 ^b^	<0.05
Ileum			
Villous height, μm	402.11 ± 29.39	424.45 ± 15.43	0.10
Crypt depth, μm	163.09 ± 13.47	153.84 ± 9.55	0.24
Villous height/crypt depth	2.52 ± 0.22 ^a^	3.09 ± 0.17 ^b^	<0.01

^1^ IUGR pigs were fed with a basal diet supplemented with unfermented milk (control) and fermented milk (treatment). Data are analyzed with an unpaired *t*-test and indicated as means ± SD, n = 6. Different superscripts within rows indicate statistical significance at *p* < 0.05.

**Table 5:** Digestive enzyme activity in the small intestine of weaned IUGR piglets supplemented with fermented milk ^1^.

Item	Control	Treatment	*p*-Value
Duodenum			
Lipase, U/g protein	75.31 ± 34.29 ^a^	120.33 ± 24.49 ^b^	<0.05
α-amylase, U/mg protein	98.09 ± 26.94 ^a^	152.35 ± 36.74 ^b^	<0.05
Sucrase, U/mg protein	44.64 ± 6.37 ^a^	87.41 ± 44.09 ^b^	<0.05
Maltase, U/mg protein	131.03 ± 58.79	198.58 ± 51.44	0.08
Jejunum			
Lipase, U/g protein	64.57 ± 17.64	77.29 ± 23.03	0.31
α-amylase, U/mg protein	88.40 ± 26.94	101.38 ± 51.44	0.59
Sucrase, U/mg protein	71.81 ± 13.23 ^a^	98.08 ± 18.62 ^b^	<0.05
Maltase, U/mg protein	192.92 ± 39.19	261.79 ± 93.08	0.14
Ileum			
Lipase, U/g protein	78.78 ± 13.96	82.84 ± 5.88	0.57
α-amylase, U/mg protein	68.60 ± 16.90	88.06 ± 19.35	0.10
Sucrase, U/mg protein	70.23 ± 26.94	95.25 ± 18.62	0.13
Maltase, U/mg protein	202.06 ± 53.89	302.23 ± 90.63	0.06

^1^ IUGR pigs in the control group and treatment group were fed with a basal diet supplemented with unfermented milk and fermented milk, respectively. Data are expressed as means ± SD, n = 6. Different superscripts within rows denote statistical significance at *p* < 0.05.

**Table 6:** Intestinal and hepatic redox status in IUGR weaned piglets supplemented with fermented milk ^1^.

Item	Control	Treatment	*p*-Value
Jejunum			
GSH, nmol/mg tissue	1.42 ± 0.34	1.62 ± 0.24	0.29
GSSG, nmol/mg tissue	0.11 ± 0.02	0.12 ± 0.02	0.46
GSH:GSSG	13.13 ± 0.44 ^a^	14.46 ± 1.05 ^b^	<0.05
MDA, nmol/mg protein	0.36 ± 0.12	0.34 ± 0.10	0.88
Ileum			
GSH, nmol/mg tissue	1.18 ± 0.12 ^a^	1.73 ± 0.20 ^b^	<0.01
GSSG, nmol/mg tissue	0.11 ± 0.02	0.12 ± 0.02	0.67
GSH:GSSG	10.69 ± 0.49 ^a^	15.14 ± 2.42 ^b^	<0.01
MDA, nmol/mg protein	0.30 ± 0.05	0.27 ± 0.05	0.45
Liver			
GSH, nmol/mg tissue	13.27 ± 0.42	15.47 ± 0.29	0.15
GSSG, nmol/mg tissue	1.06 ± 0.02	1.05 ± 0.02	0.47
GSH:GSSG	12.15 ± 1.64 ^a^	15.21 ± 1.52 ^b^	<0.01
MDA, nmol/mg protein	1.50 ± 0.39 ^a^	0.86 ± 0.07 ^b^	<0.01

^1^ IUGR pigs in the control group and treatment group were fed with a basal diet supplemented with unfermented milk and fermented milk, respectively. Data are expressed as means ± SD, n = 6. Different superscripts within rows mark statistical significance at *p* < 0.05. GSH, Glutathione; GSSG, glutathione disulfide; MDA, malondialdehyde.

## Data Availability

The data presented in this study are included in the article and Supplementary Materials.

## Supplementary Materials

The following supporting information can be downloaded at https://www.mdpi.com/article/10.3390/ani15101367/s1. Table S1: Formula and conditions for milk fermentation. Table S2: Concentrations of free amino acids (μmol/L) in fermented and unfermented milk. Table S3: Concentrations of total amino acids (mmol/L) in fermented and non-fermented milk. Table S4: Ingredient and chemical composition of the basal diet (as-fed basis). Table S5: Primer sequences.
