# Supplementary material for: Dietary Supplementation with Fermented Milk Improves Growth Performance and Intestinal Functions in Intrauterine Growth-Restricted Piglets
Source: Animals (Basel). 2025 May 9;15(10):1367. doi: 10.3390/ani15101367 (PMC12108382; doi:10.3390/ani15101367)
Supplement: Supplementary file 1 [file animals-15-01367-s001.zip › animals-3572700-supplementary.pdf]

Table S1. Formula and conditions for milk fermentation.

Item	Parameters
Milk powder, kg	15
Glucose, kg	5
Inoculant mixture[1], g	2
Sterile water, kg	80
Fermentation time, h	13.5
Temperature	～30℃

[1] The inoculant mixture was composed of *P. pentosaceus*, *L. plantarum*, *B. subtilis* and *S. cerevisae*.

Table S2. Concentrations of free amino acids (μmol/L) in fermented and unfermented milk[1].

Item	Before fermentation	After fermentation	*P*-value
Alanine	42.03 ± 0.52[a]	60.07 ± 7.10[b]	< 0.05
Arginine	6.33 ± 1.73	11.67 ± 4.50	0.19
Asparagine	3.86 ± 0.17	8.64 ± 2.77	0.08
Aspartate	35.03 ± 0.69[a]	68.04 ± 12.82[b]	< 0.05
Glutamine	18.01 ± 0.35	18.12 ± 2.08	0.83
Glutamate	287.23 ± 4.33[a]	128.01 ± 16.97[b]	< 0.05
Glycine	223.03 ± 8.83	227.01 ±14.55	0.73
Histidine	32.03 ± 0.69[a]	79.95 ± 9.70[b]	< 0.01
Isoleucine	8.37 ± 1.73[a]	19.04 ± 5.02[b]	< 0.05
Leucine	12.95 ± 2.77[a]	39.95 ± 6.93[b]	< 0.01
Lysine	41.01 ± 3.64[a]	116.04 ± 20.78[b]	< 0.01
Methionine	1.87 ± 0.35[a]	7.02 ± 1.73[b]	< 0.01
Phenylalanine	6.03 ± 0.52[a]	21.04 ± 2.77[b]	< 0.01
Serine	17.01 ± 0.87	21.97 ± 3.64	0.15
Threonine	5.43 ± 0.17	9.98 ± 5.37	0.29
Tryptophan	4.70 ± 0.17	5.94 ± 1.04	0.14
Tyrosine	5.63 ± 0.52[a]	34.02 ± 7.97[b]	< 0.01
Valine	16.03 ± 0.17	29.95 ± 5.54	0.06
β-Alanine	4.30 ± 0.17	2.75 ± 1.21	0.29
Citrulline	4.63 ± 0.69[a]	23.98 ± 5.02[b]	< 0.01
Ornithine	138.17 ± 2.77[a]	156.04 ± 8.83[b]	< 0.05
Taurine	36.03 ± 2.25	35.95 ± 2.42	0.92

[1]Data are expressed as means ± SD of triplicates. Values with different superscripts in a row are significant at $P < 0.05$ determined by an unpaired-test.

Table S3. Concentrations of total amino acids (mmol/L) in fermented and non-fermented milk[1].

Item	Before fermentation	After fermentation	*P*-value
Alanine	8.67 ± 1.39	7.70 ± 0.35	0.12
Arginine	3.86 ± 0.87[a]	3.06 ± 0.17[b]	< 0.05
Asparagine+Aspartate[2]	16.40 ± 0.87	15.53 ± 0.52	0.17
Glutamine+Glutamate[3]	33.71 ± 0.69	35.84 ±1.56	0.15
Glycine	16.77 ± 4.50[a]	12.74 ± 0.35[b]	0.05
Histidine	3.96 ± 0.69	3.27 ± 0.17	0.08
Isoleucine	10.54 ± 1.73[a]	8.50 ± 0.35[b]	< 0.05
Leucine	21.37 ± 1.39	19.50 ± 0.87	0.08
Lysine	17.20 ± 0.52[a]	12.11 ± 0.17[b]	< 0.05
Methionine	7.03 ± 0.17	6.93 ± 0.35	0.65
Phenylalanine	6.91 ± 0.35[a]	6.13 ± 0.35[b]	< 0.05
Serine	12.67 ± 1.73[a]	10.90 ± 0.35[b]	0.05
Threonine	7.76 ± 0.52	7.11 ± 0.35	0.14
Tryptophan	1.48 ± 0.17	1.73 ± 0.17	0.06
Tyrosine	6.03 ± 0.52	5.56 ± 0.17	0.07
Valine	13.53 ± 0.69	12.96 ± 0.52	0.43

[1]Data are expressed as the means ± SD of triplicates. Different superscripts within rows mark statistical significance at $P < 0.05$.

[2]Asparagine in the protein lysate was detected in the form of aspartate.

[3]Glutamine in the protein lysate was detected in the form of glutamate.

Table S4. Ingredient and chemical composition of the basal diet (as-fed basis).

**Ingredient**	**Composition, %**
Corn	43.64
Extruded corn	20.00
Soybean meal	6.40
Fermented soybean meal	6.00
Extruded soybean	8.00
Fish meal	4.00
Whey powder	6.50
Soybean oil	2.00
L-Lysine (99%)	0.15
Sodium chloride	0.40
Calcium carbonate	0.53
Dicalcium phosphate	1.38
Premix[1]	1.00
Total	100.00
**Nutrient level[2]**	
Digestible energy, MJ/kg (calculated value)	14.67
Crude protein (measured value)	19.01
Lysine	1.20
Methionine	0.46
Threonine	0.53
Tryptophan	0.26

[1]The composition of the premix was as follows (per kg of the diet): 10000 IU of vitamin A, 1500 IU of vitamin D_3, 40 IU of vitamin E, 2 mg of vitamin B_1, 4 mg of vitamin B_2, 0.04 mg of vitamin B_{12}, 30 mg of niacin, 13 mg of pantothenic acid, 0.16 mg of biotin, 0.6 mg of folic acid, 120 mg of copper, 100 mg of iron, 100 mg of zinc, 30 mg of manganese, 0.2 mg of iodine, and 0.4 mg of selenium.

[2]The digestible energy of the diet was calculated, while the contents of other nutrients in the diet were measured according to respective procedures.

Table S5. Primer sequences.

Gene	GenBank ID	Primer sequence (5′→3′)	Product length
IL-1β	XM_003355629.4	F: AGAAGTCTCACGAACAGCCA<br>R: AAAACGGACATGCCTAGGGA	83
IL-6	NM_001317081.1	F: GATTGTGGGCAGTGGAATCC<br>R: AAGACCCTCCTTCATTGGCA	146
IL-8	NM_001164649.1	F: GACGTCACCCTGATCATTGC<br>R: TTGTCAACAGCAAAGCCTCC	242
TNF-α	XM_021069181.1	F: AGAGAAGCTCCTAGGGGTCA<br>R: AGGGTGGAGATGCATGTGAA	194
GAPDH	NM_001206359.1	F: CTCGGAGTGAACGGATTTGG<br>R: AGTGGAGGTCAATGAAGGGG	109
